# Identification of molecular signatures involved in radiation-induced lung fibrosis

**DOI:** 10.1007/s00109-018-1715-9

**Published:** 2018-11-07

**Authors:** Hee Jin, Ga-Young Kang, Seulgi Jeon, Jin-Mo Kim, You Na Park, Jaeho Cho, Yun-Sil Lee

**Affiliations:** 1 Graduate School of Pharmaceutical Sciences, Ewha Womans University, Seoul, 120-750 South Korea; 2 Department of Radiation Oncology, Yonsei University Health System, Seoul, 120-749 South Korea

**Keywords:** Irradiation, Lung fibrosis, Epithelial-mesenchymal transition, *gtse1*, *fgl1*

## Abstract

**Abstract:**

In radiotherapy, radiation (IR)-induced lung fibrosis has severe and dose-limiting side effects. To elucidate the molecular effects of IR fibrosis, we examined the fibrosis process in irradiated mouse lung tissues. High focal IR (90 Gy) was exposed to a 3-mm volume of the left lung in C57BL6 mice. In the diffused irradiation, 20 Gy dose delivered with a 7-mm collimator almost covered the entire left lung. Histological examination for lung tissues of both irradiated and neighboring regions was done for 4 weeks after irradiation. Long-term effects (12 months) of 20Gy IR were compared on a diffuse region of the left lung and non-irradiated right lung. Fibrosis was initiated as early as 2 weeks after IR in the irradiated lung region and neighboring region. Upregulation of *gtse1* in both 90Gy-irradiated and neighboring regions was observed. Upregulation of *fgl1* in both 20Gy diffused irradiated and non-irradiated lungs was identified. When *gtse1* or *flg1* was knock-downed, TGFβ or IR-induced epithelial-mesenchymal transition was inhibited, accompanied with the inhibition of cellular migration, suggesting fibrosis responsible genes. Immunofluorescence analysis using mouse fibrotic lung tissues suggested that fibrotic regions showed increased expressions of Gtse1 and Fgl1, indicating novel molecular signatures of *gtse1*and *fgl1* for IR-induced lung fibrosis. Even though their molecular mechanisms and IR doses or irradiated volumes for lung fibrosis may be different, these genes may be novel targets for understanding IR-induced lung fibrosis and in treatment strategies.

**Key messages:**

- Upregulation of *gtse1* by 90Gy focal irradiation and upregulation of *fgl1* by 20Gy diffused irradiation are identified in mouse lung fibrosis model.
- Gtse1 and Fgl1 are involved in radiation or TGFβ-induced epithelial-mesenchymal transition.
- Radiation-induced fibrotic regions of mouse lungs showed increased expressions of Gtse1 and Fgl1.
- Gtse1 and Fgl1 are suggested to be novel targets for radiation-induced lung fibrosis.

**Electronic supplementary material:**

The online version of this article (10.1007/s00109-018-1715-9) contains supplementary material, which is available to authorized users.

## Introduction

Radiation (IR)-induced pulmonary fibrosis is a frequently occurred complication from radiotherapy threatening the health and life of patients [1, 2]. The time of onset and severity of lung fibrosis after radiotherapy depend on many factors, including the volume of irradiated parenchyma, dose of absorbed IR, and number of fractions [3–5]. Despite clinical evidence of lung injury due to IR, the molecular mechanisms underlying the effect of IR, especially those focusing on lung fibrosis, have not been clearly identified.

The goal of high-dose per fraction irradiation (HDFR), such as stereotactic body radiotherapy (SBRT), is to administer a markedly higher dose to the treatment target volume without damaging the surrounding normal tissue. The targeting accuracy of IR to the tumor by SBRT produces reduced damage to surrounding normal tissue. It is feasible to use HDFR without inducing significant acute and late IR-induced toxicity with SBRT. However, there are still concerns of late toxicity following high-dose treatment. Because large doses of IR are delivered to the target volume, adjacent normal tissue damage may not compromise the benefits of HDFR for better control because underlying radiobiological mechanisms of damage by large dose per fractions remain the same.

We have previously developed a mouse model simulating clinical SBRT and have used the model to validate the induction of lung fibrosis by high-dose IR [6]. We also attempted to understand the biological changes occurring in the process of lung tissue damage including cell death after SBRT. In addition, we identified responsible genes and proteins for IR-induced lung damage [7]. In this study, to identify molecular signatures for IR-induced lung fibrosis, we further examined the fibrosis process at 4 weeks in non-irradiated neighboring regions as well as irradiated region from mouse lung tissues after HDFR (90 Gy). The regimen was similar to that used for human therapy reflecting the understanding of the clinically related HDFR-mediated normal cell damage like fibrosis. Long-term effects (12 months) on diffuse region of the left lung and non-irradiated right lung after 20Gy IR which dose ranges (15–20 Gy) are frequently used as IR-induced lung fibrosis model [8–11] were also compared.

## Materials and methods

### Antibody and reagents

Protein levels were detected using commercial antibodies to the following: GTSE1 (Thermo Scientific, Waltham, MA, USA or Sigma-Aldrich, St. Louis, MO, USA), FGL1 (Thermo Scientific or Sigma-Aldrich); α-SMA (Sigma-Aldrich); Twist (Abcam, Cambridge, UK), Matrix metallopeptidase 12 (MMP12; Abcam), Gapdh (Abcam); Fibronectin (BD Biosciences, Santa Clara, CA, USA); and Twist (Santa Cruz Biotechnology, Santa Cruz, CA, USA), β-Actin (Santa Cruz). Predesigned small interfering (si) RNAs for human *gtse1* and *fgl1* and a negative control siRNA were purchased from Bioneer (Daejeon, Republic of Korea). Transforming growth factor-beta 1 (TGF-β1) was purchased from BD Biosciences.

### Cell culture and transfection

A549 human lung adenocarcinoma cells and L132 human pulmonary epithelial cells were supplied from American Type Culture Collection (ATCC, Manassas, VA, USA). All cells were cultured in RPMI medium supplemented with 10% fetal bovine serum and 1% penicillin-streptomycin at 37 °C in humidified 5% CO_2_ incubator. Transient transfection of all cell types used Lipofectamine 2000 (Invitrogen, Carlsbad, CA, USA) as instructed by the manufacturer.

### Irradiation

Five adult male C57BL/6 mice (Central Lab Animal Inc., Seoul, Korea) were housed per each cage (10-week-old). Total 60 male mice were randomly divided into four groups. Two groups were for 90Gy experiment (control and 90Gy focal irradiated groups) and two groups were for 20Gy experiment (control and 20Gy irradiated groups). As the clinical SBRT condition, we selected a 3-mm collimator to administer a 90Gy dose to the central area of the left lung. For low-dose diffused irradiation conditions, we delivered a 20Gy dose with a 7-mm collimator, which almost covered the entire left lung. In the mice that underwent 90Gy irradiation, focal irradiated and neighboring tissues were separately isolated (Supplementary Fig. S1b). In the mice that underwent 20Gy irradiation, the whole left lungs were used for irradiated tissues and non-irradiated right lungs were also used. Control lungs were isolated from the age-matched control mice. Radiation was administered with an X-RAD 320 (Precision, North Branford, CT) equipped with a collimator system that consisted of 5-cm-thick copper to focus the radiation beams. X-ray beam dose rate was 19.7 cGy/s. Detailed methods are described previously [12]. In the case of cells, they were exposed to γ-rays using a ^137^Cs γ-ray source (Atomic Energy of Canada) with a dose rate of 3.81 Gy/min.

### Microarray experiment, RNA isolation, and quantitative RT-PCR (qPCR)

Total RNA from the mouse lung tissues was prepared using the Easy-SpinTM total RNA extraction kit according to the manufacturer’s instructions (iNtRON Biotechnology, Seoul, Republic of Korea). RNAs from 3 mice at each time point were pooled to exclude experimental bias. Detailed methods are described in the Supplementary information. For quantitative RT-PCR, total RNA was isolated from lung tissues of experimental mice or cells at each time points post radiation exposure using TRIzol® reagent (Qiazen, Valencia, CA, USA). RNA purity and concentration were measured with a Nanodrop. RNA was reverse transcribed using a ReverTra Ace® qPCR RT Kit (TOYOBO, Kita-ku, Osaka, Japan) following the manufacturer’s protocol and PCR was performed to assess expression of the candidate genes using primers designed for mouse mRNA sequences. Also, the expression of mRNAs was assessed by real-time PCR using SYBR Green PCR Master Mix kit (Invitrogen, Carlsbad, CA, USA) with an ABI 7300 real-time PCR thermal cycler (Applied Biosystems, Foster City, CA, USA). The 2^−ΔΔCt^ method could be used to analyze the relative changes in gene expression from real-time quantitative PCR experiments. The total reaction volume is 20 μL. Reaction conditions started with enzyme activation at 95 °C for 10 min, followed by 40 cycle of 95 °C for 15 s, 58 °C for 30 s, and 72 °C for 45 s. Primer sequences for qRT-PCR are listed in Supplementary Table S1.

### Immunoblotting

For immunoblotting, cells were lysed with immune precipitation buffer. Protein concentration was determined by the Bradford method (Bio-Rad, Hercules, CA, USA). The samples were boiled for 5 min and an equal amount of protein was analyzed by SDS-PAGE (6–15%) using standard conditions. HRP activity was measured using enhanced chemiluminescence (EzWestLumi, Taito-ku, Tokyo, Japan). Protein band intensity was visualized on ChemiDoc (Bio-Rad) and quantified using Image J software 1.45 (National Institutes of Health, Bethesda, MD, USA).

### Histological and immunohistochemical analysis

Lung tissues were formalin-fixed, paraffin-embedded, and sections were prepared for standard hematoxylin/eosin (H&E) staining, Masson trichrome (Sigma-Aldrich) collagen staining, and picro-sirius red (American MasterTech Scientific, Lodi, CA, USA) staining [13].

### Wound healing assay

For monolayer wound-healing assays, cells were plated in a 60-mm dish and at 100% confluence, two parallel wounds of 1 mm were made using a SPLScar (SPL, Gyeonggi-do, Korea). Wound size after 0, and 48 h was measured using a light microscope (Cal Zeiss, Oberkochen, Germany) in three independent experiments.

### Immunofluorescence

Cells were fixed with 4% paraformaldehyde followed by permeabilization with 0.1% sodium citrate plus 0.1% Triton X-100. For dual immunofluorescence staining, cells were incubated with Fibronectin (1:200) or Twist (1:200) and GTSE1 (1:100) or FGL1 (1:100) antibodies for 1 h at room temperature. The cells were incubated with Alexa 568-labeled anti-rabbit (1:1000) and Alexa 488-labeled anti-mouse (1:1000) secondary antibodies. For lung tissue immunofluorescence assays, de-paraffinized slides were boiled in 0.1 M citrate buffer (pH 6.0) for 20 min and slides were co-immunostained with α-SMA (1:200), GTSE1 (1:200), and FGL1 (1:200) overnight at 4 °C. Nucleus was counterstained with DAPI (Sigma-Aldrich) and stained cells were imaged using a Zeiss Apotome (Cal Zeiss).

### Statistical analysis

Data were analyzed using GraphPad Prism 5.0 (GraphPad Software Inc., San Diego, CA). Statistical difference compared with control group was determined by original one-way ANOVA with Dunnett’s multiple comparisons test and Student’s *t* test; *p* values < 0.05 were considered significant.

## Results

### Development of lung fibrosis after 90Gy HDFR

**Fig. 1:**
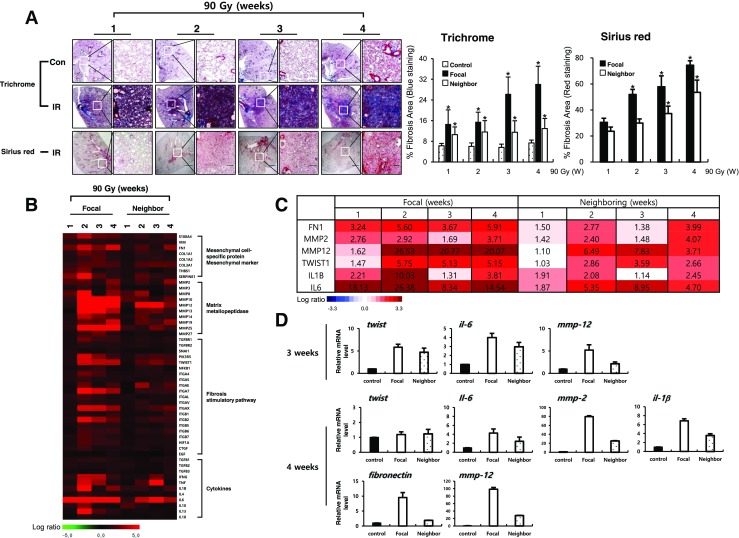
Fibrotic changes of focal and neighboring regions after focal high-dose radiation (90 Gy). **a** Masson’s trichrome (upper) and Sirius red (bottom) from animals in the non-irradiated control (Con) and irradiated (90Gy IR) groups. Graphs represent quantification of fibrosis score in focal lung regions and neighboring lung regions. The arrows indicate the focally irradiated area. Magnification, × 1.25 and × 100; scale bar, 50 μm (*n* = 3, mean ± SD, *p < 0.05 vs. compared to the age-matched unirradiated control group (Trichrome), *p < 0.05 vs. compared to the corresponding 1 week group (Sirius red), one-way ANOVA). **b** Heat-map representing the differentially expressed fibrosis-related genes by focal high-dose radiation in both irradiated and neighboring regions at the indicated time points. Each column represents pooled lung tissue RNA samples from three mice at each time point to exclude experimental bias. The expression ratio color scale ranges from red (high) to green (low), as indicated by the scale bar with log 2 units. **c** List of fibrosis-related genes with a fold ratio > 2 or < 0.5 (for up and downregulation, respectively) compared to the control. **d** Quantitative RT-PCR using focal irradiated and non-irradiated neighboring lung tissue from three individual mice at the indicated time points. Each mRNA expression was normalized to *gapdh* (*n* = 3, mean ± SD)

Significant abnormalities were observed subsequent to the focused dose radiation in the H&E-stained sections collected at different times (Supplementary Fig. S2a) [14]. Collagen deposition assays of Masson’s trichrome staining to visualize the blue-colored collagen deposition or sirius red staining to visualize the red-colored collagen deposition after IR revealed fibrotic changes that began in the focal irradiated area, with peak collagen deposition evident 4 weeks after IR. Fibrotic changes were also observed in the tissue neighboring the focally irradiated areas of the lungs, similar to focally irradiated regions. However, fibrosis intensities were much weaker in non-irradiated neighboring tissues than focal irradiation regions. Without IR, age-related collagen deposition was not occurred in lung tissues until 4 weeks (Fig. 1a and Supplementary Fig. S2a). Hydroxyproline staining data also indicated the development of fibrosis in both focal and neighboring tissues (Supplementary Fig. S2c). The cDNA microarray data suggested that fibrosis-related genes involving mesenchymal markers, matrix metallopeptidase, fibrosis stimulatory pathways, and cytokines were changed according to fibrosis development. These phenomena were also detected in neighboring tissues (Fig. 1b and Supplementary Fig. S3). Especially, expressions of *fibronectin*, *mmp2*, *mmp12*, *twist1*, *il1β*, and *il6* were markedly increased in focally irradiation regions 3 or 4 weeks after IR in microarray data. The findings were confirmed by qRT-PCR, which also correlated well in neighboring tissues (Fig. 1c).

### Development of lung fibrosis after 20Gy diffused whole-lung irradiation

**Fig. 2:**
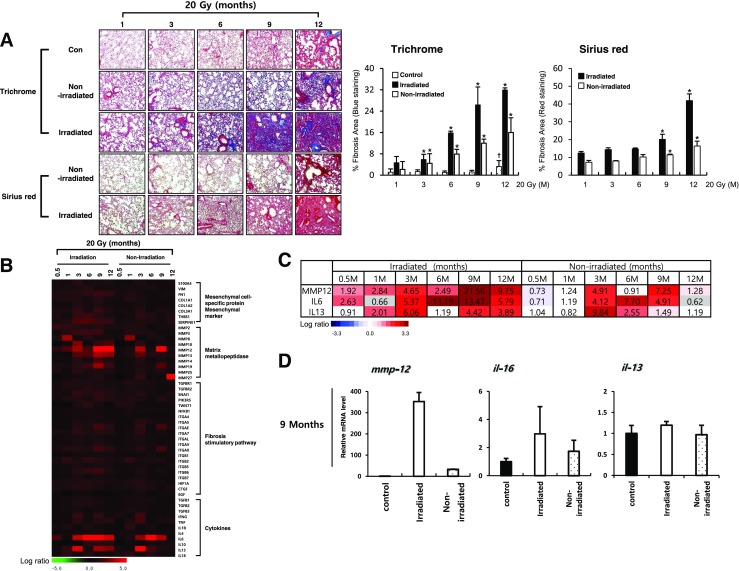
Fibrotic changes of irradiated and non-irradiated regions by diffused irradiation (20 Gy). **a** Masson’s trichrome (upper) and Sirius red (bottom) from animals in the non-irradiated control (Con) and 20Gy irradiated groups. Graphs represent quantification of fibrosis score in irradiated left lung regions and non-irradiated right lung regions. Magnification, × 1.25 and × 100; scale bar, 50 μm (*n* = 3, mean ± SD, *p < 0.05 vs. compared to the age-matched unirradiated control group (Trichrome), *p < 0.05 vs. compared to the corresponding 1-week group (Sirius red), †*p* < 0.05 vs. compared to the 1-week unirradiated control group, one-way ANOVA). **b** Heat-map representing the differentially expressed fibrosis-related genes by diffused irradiation in both irradiated left and non-irradiated right lungs. Each column represents pooled lung tissue RNA samples from three mice at each time point to exclude experimental bias. The expression ratio color scale ranges from red (high) to green (low), as indicated by the scale bar with log 2 units. **c** List of fibrosis-related genes with a fold ratio > 2 or < 0.5 (for up and downregulation, respectively) compared to the control. **d** Quantitative RT-PCR using focal irradiated and non-irradiated neighboring lung tissue from three individual mice at the indicated time points. Each mRNA expression was normalized to *gapdh* (*n* = 3, mean ± SD)

We also examined fibrosis development by diffused irradiation, 20 Gy (7 mm) to the entire left lungs of mice. A significant amount of lung fibrosis was observed, with peak collagen deposition at 12 months after IR. There were more fibrotic areas following 90Gy HDFR than 20Gy. Interestingly, collagen deposition was also observed in non-irradiated right lung tissues after IR, although fibrosis intensity was much weaker than irradiated left lungs. Age-dependent collagen deposition was detected at later time point of 12 months; however, IR more facilitated the fibrosis development in both irradiated and non-irradiated regions (Fig. 2a and Supplementary Fig. S2b). Hydroxyproline staining data also indicated the development of fibrosis in both irradiated left lung and non-irradiated right lung tissues (Supplementary Fig. S2d). Fibrosis-related genes from the microarray data re-confirmed by qRT-PCR revealed several differences from HDFR (Fig. 2b and Supplementary Fig. S3). Only *mmp12*, *il6*, and *il13* genes were altered in both irradiated left lungs and non-irradiated right lungs (Fig. 2c). When TGFβ-related gene expressions were extracted from the microarray data, no dramatic alteration was observed in both 90Gy and 20Gy irradiated lungs, except for slight alterations of TGFβ, TGFβ-receptor 1 (TGFBR1), and Smad 6 (Supplementary Fig. S4a).

### Selection of differentially expressed genes

**Fig. 3:**
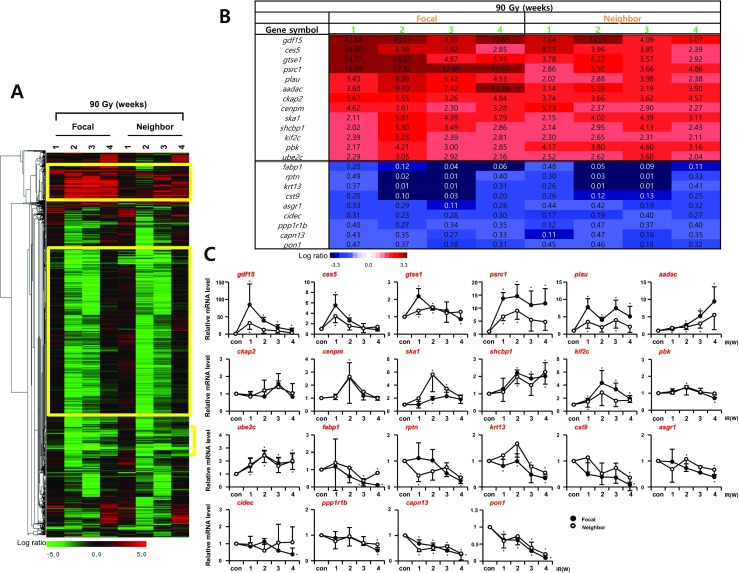
Temporal gene expression patterns in focal irradiated and neighboring lung regions by 90 Gy. **a** Microarray data from mouse lungs were obtained at indicated time points after exposure to 90 Gy. The expression ratio color scale ranges from red (high) to green (low), as indicated by the scale bar with log 2 units. **b** List of candidate genes with a fold ratio > 2 or < 0.5 (for up and downregulation, respectively) compared to the control. **c** Confirmation of candidate genes by quantitative RT-PCR using the lungs from three individual mice. Each mRNA expression was normalized to *gapdh* (*n* = 3, mean ± SD, *p < 0.05 vs. corresponding control, one-way ANOVA)

The temporal changes in gene expression of both focal irradiated and neighboring tissues following HDFR were hierarchically clustered. One week after HDFR, there were no noticeable changes in gene expression. However, at 2 and 3 weeks, expression of a predominant number of genes was downregulated and then restored to the control level at 4 weeks after IR. In addition to the downregulated pattern, we also identified other temporally upregulated genes, especially in the focal area of the lungs that received 90Gy IR (Fig. 3a). Interestingly, this major pattern of gene expression was also observed in the neighboring lung areas but was not observed in the lungs irradiated with 20 Gy. To identify genes responsive to 90 Gy in both focal irradiated and neighboring tissues, we selected genes presenting greater than twofold change in expression compared to the control group at every time points. The downregulated genes (*fabp1*, *rptn*, *krt13*, *cst9*, *asgr1*, *cidec*, *ppp1r1b*, *capn13*, and *pon1*) and upregulated genes (*gdf15*, *ces5*, *gtse1*, *psrc1*, *plau*, *aadac*, *ckap2*, *cenpm*, *ska1*, *shcbp1*, *kif2c*, *pbk*, and *ube2c*) were selected as candidate HDFR responsive genes in both focal irradiated and neighboring tissues (Fig. 3b), which were confirmed by qRT-PCR (Fig. 3c).

**Fig. 4:**
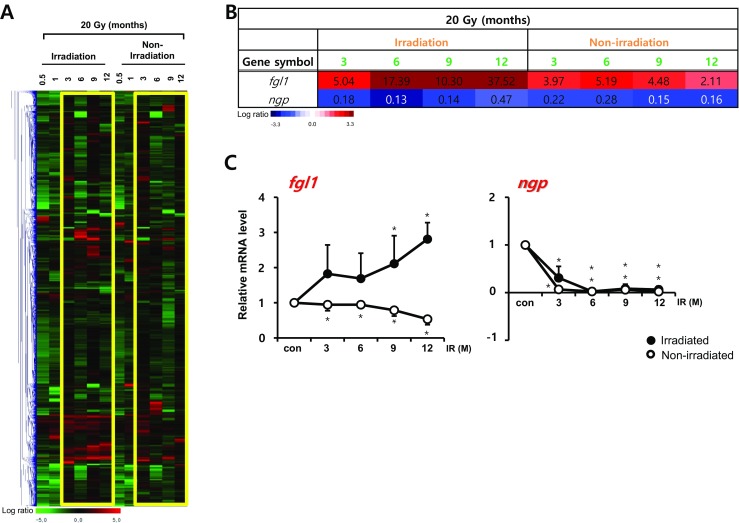
Temporal gene expression patterns in irradiated and non-irradiated lung tissue by diffused irradiation (20 Gy). **a** Microarray data from mouse lungs were obtained at indicated time points after exposure to 20 Gy. The expression ratio color scale ranges from red (high) to green (low), as indicated by the scale bar with log 2 units. **b** List of candidate genes with a fold ratio > 2 or < 0.5 (for up and downregulation, respectively) compared to the control. **c** Confirmation of candidate genes by quantitative RT-PCR using lungs from three individual mice. Each mRNA expression was normalized to *gapdh* (*n* = 3, mean ± SD, *p < 0.05 vs. corresponding control, one-way ANOVA)

We also compared temporal patterns of gene expression in diffused irradiated left lungs and non-irradiated right lung tissue with 20 Gy (Fig. 4a). Unlike the HDFR microarray data, gene expression patterns of irradiated and non-irradiated regions were not similar. When we selected more than twofold changes in expression compared to the control group at every time points, only two genes (the downregulated gene, *ngp*, and the upregulated gene, *fgl1*; the latter not observed in lungs irradiated with HDFR) were selected as candidate responsive genes for fibrosis in both 20Gy irradiated and non-irradiated lung tissue (Fig. 4b). The results were confirmed by qRT-PCR (Fig. 4c). Gene of *gtse1*, selected from the 90Gy irradiated lung tissues, and gene of *fgl1*, selected from 20Gy irradiated lung tissues, were also overexpressed in microarray data of other dose irradiated lungs, although the intensity was weaker (Supplementary Fig. S4b), suggesting both genes are regarded as IR-responsible genes regardless of IR doses.

### Genes responsible for IR-induced epithelial-mesenchymal transition (EMT)

**Fig. 5:**
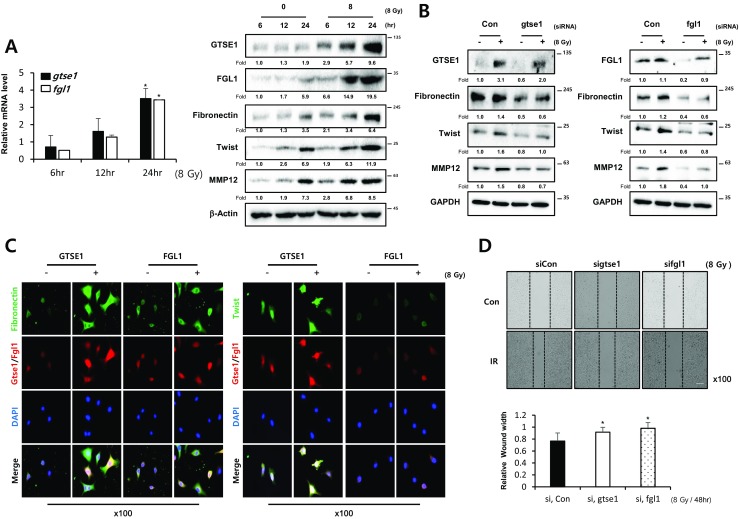
Radiation induces EMT through *gtse1* or *fgl1*. **a** Expression of GTSE1 and FGL1 in L132 cells was examined by qRT-PCR (left) and Western blotting (right) analysis after 8Gy radiation (IR) at indicated time points. **b** Western blot analysis in L132 cells after transfection of siRNA *gtse1* or *fgl1* with or without 8Gy radiation (IR). The fold increase of each time point mRNA was normalized with the non-irradiated cells (*n* = 3, mean ± SD, *p < 0.05 vs. corresponding control, paired sample *t* test) and protein levels were quantified using Image J software, and data are expressed as the fold change relative to the negative control. **c** Co-immunofluorescence staining of Fibronectin or Twist (green) and GTSE1 (red) or FGL1 (red) after 24 h of irradiation (8 Gy) in A549 cells. Nuclei were counterstained with DAPI (blue). **d** Wound healing assays in A549 cells after transfection of siRNA *gtse1* or *fgl1* with or without irradiation (8 Gy). Cell movement into wound was shown at 48h post-scratch. Graphs represent relative wound width (mean ± SD, *p < 0.05 vs. corresponding control, paired sample *t* test). All representative photomicrographs magnification, × 100; bar, 50 μm

To determine the genes responsible for IR-induced lung fibrosis, siRNA of each gene that showed relatively higher expression or lower expression in microarray data was transfected to L132 cells and RT-PCR analysis for EMT-related genes such as *E-cadherin* and *snail* was performed after treatment of TGFβ (data not shown). The *gtse1* and *fgl1* were identified as candidate genes from the results, and these two genes (both mRNA and protein) responded to both IR (8 Gy) and TGFβ (5 ng/ml), accompanied with increased Snail, Twist, MMP12 and Fibronectin, and reduction of ZO-1 (Fig. 5a and Supplementary Fig. S5a). Altered expression of EMT markers by IR or TGFβ was partially restored in terms of protein level and mRNA levels of L132 normal lung cells (Fig. 5b and Supplementary Fig. S5b). TGFβ-mediated morphological changes were restored by siRNA of gste1 or fgl1 (Supplementary Fig. S5c). The results suggested the involvement of Gste1 and Fgl1 in IR or TGFβ-mediated EMT. Co-expression of Fibronectin or Twist with Gtse1 or Fgl1 after IR or TGFβ treatment suggested the involvement of these genes in EMT processing (Fig. 5c and Supplementary Fig. S5d). Moreover, knockdown of gtse1 or fgl1 inhibited IR or TGFβ-mediated migration (Fig. 5d and Supplementary Fig. S5e) even though knockdown of gtse1 or fgl1 did not induce any increased cell death after IR or TGFβ (Supplementary Fig. S6).

### Overexpression of Gtse1 and Fgl1 in IR-induced fibrotic lung tissues

**Fig. 6:**
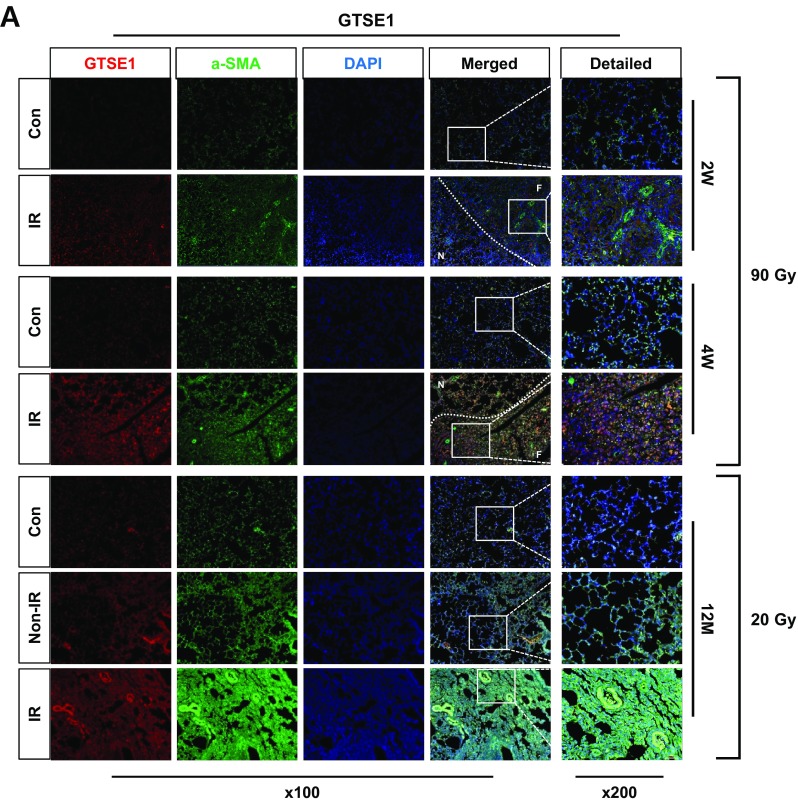

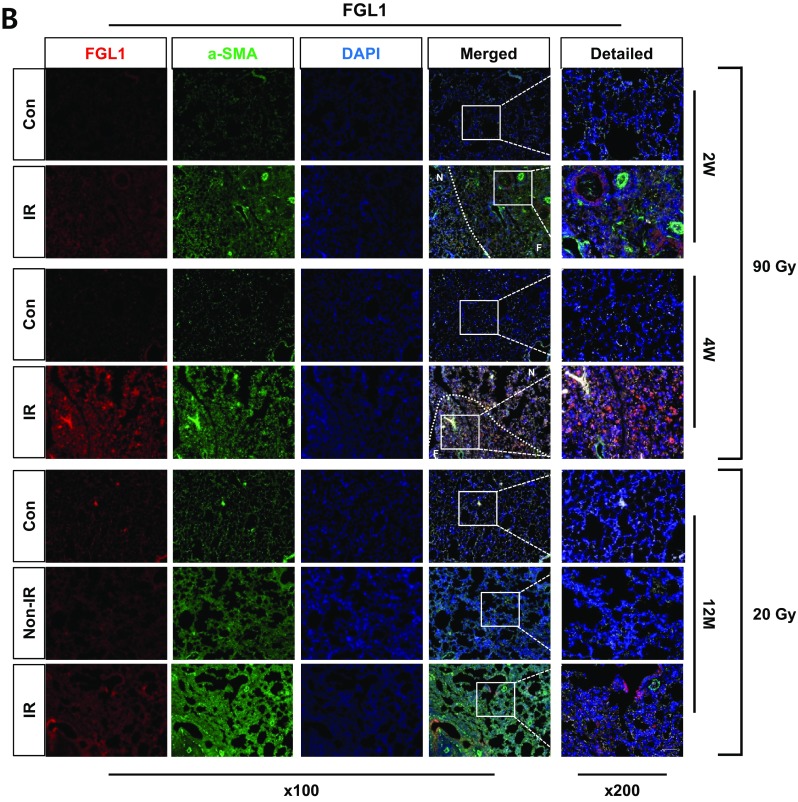
Overexpression of GTSE1 or FGL1 in IR-induced lung fibrotic tissues. **a**, **b** Co-immunofluorescence staining of α-SMA (green) and GTSE1/FGL1 (red) after focal high-dose (90 Gy) and diffused whole-lung irradiation (20 Gy) at each time point. Lung tissue sections from three mice were used. Magnification, × 100 and × 200; scale bar, 50 μm. (F: focal irradiated region, N: neighboring region)

Immunofluorescence data of GTSE1 and FGL1 with co-staining of α-SMA demonstrated the increased expression in fibrotic regions of lung tissues after 20 Gy or 90 Gy. Gtse1 expression in accompanied with higher expression of α-SMA was clearly shown in non-irradiated neighboring tissues, as well as 90Gy focal irradiated regions, when compared with the age-matched unirradiated control mice at 2 and 4 weeks after IR. Moreover, its increased expression was also observed in the case of 20Gy. Fgl1 expression was dominant in 20Gy irradiated left lung tissues and in non-irradiated right lungs, when compared with the age-matched unirradiated control mice. IR with 90 Gy also increased Fgl1 expression in both focal irradiated and neighboring tissues (Fig. 6a and b).

## Discussion

Our results using SBRT mouse model demonstrate that non-irradiated neighboring regions had a similar gene expression pattern to the pattern observed in the focally irradiated lung regions. Moreover, visual confirmation and changes in gene expression provide evidence of the development of fibrosis even in non-irradiated neighboring lung regions. The fibrotic changes of the boundary regions were weaker than the focally irradiated area of the lung. Moreover, fibrosis-related genes identified in the focally irradiated areas were similarly evident in neighboring non-irradiated lung regions. Comparison to the fibrosis after the left lung diffused 20Gy irradiation revealed that the development of fibrosis in the non-irradiated right lung was also affected, even though the potency was much weaker than HDFR.

Our animal models reflect focal and high-dose irradiation of human SBRT. This study is a first trial for the investigation of SBRT-related gene expression patterns in mice during lung fibrosis. Because lung fibrosis by IR was mainly in neighboring regions of irradiated tumor tissues, we also investigated non-irradiated neighboring lungs. Down and upregulated genes were identified, which regarded as candidate HDFR responsive genes because of their similar expression patterns in focally irradiated and neighboring regions. When we compared the gene expression by 20Gy diffused irradiation, two genes responded similarly in both irradiated left lung and non-irradiated right lung. When we screened using HDFR responsive genes and 20Gy responded genes to ascertain whether they were involved in IR or TGFβ-mediated EMT in cell system, *gtse1* and *fgl1* were identified as candidate genes for regulation of EMT. GTSE1, which was identified from 90Gy focal irradiated lungs, is specifically expressed during S and G2 phases of the cell cycle [15, 16]. It is a microtubule-associated protein that interacts with microtubules or PLK1 [17]. It also responds to DNA damage and inhibits apoptosis. GTSE1 inhibition significantly decreased tumor growth in vitro and in vivo, and suppressed migration and invasion in vitro [18]. Our co-staining data of GTSE1 and Ki67 revealed that fibrotic regions showed high expression of Ki67 accompanied with GTSE1 overexpression in 90Gy focal irradiated lung tissues (Supplementary Fig. S7). The *fgl1* (fibrinogen-like protein 1 [FREP1], also termed hepassocin) was identified from 20Gy diffused irradiated lungs. FGL1 is a hepatocyte secreted protein containing a fibrinogen-related domain in its C-terminal portion [19]. The enhancement of FGL1 levels was regulated by IL-6 [20, 21] and it participates in the development of non-alcoholic fatty liver disease, hepatocellular carcinomas, and hepatocyte mitogenic activity [22–26]. However, there are no reports of the involvement of any of these genes in EMT or fibrosis. This study for the first time suggests GTSE1 and FGL1 as regulators of EMT and cellular migration.

HDFR and low-dose diffused radiation showed different fibrosis development and molecular events and regulators may not be same. GTSE1 was selected from 90Gy HDFR lung and FGL1 from diffused irradiated lung. However, in vitro cell system, these two genes were all responded (showed the increased expression) by both IR and TGFβ, suggesting involvement in common fibrosis pathways regardless of fibrosis inducers. Moreover, immunostaining using lung tissues indicated that GTSE1 and FGL1 were increased at fibrotic regions regardless of focal high-dose and diffused irradiation. Since IR slightly induced mRNA of TGFβ, TGFBR1, and Smad 6 especially in 90Gy irradiated lungs, TGFβ-mediated direct or indirect EMT mechanisms by IR may involve in expression of these two gene expressions during lung fibrosis. Another interesting finding is that upregulation of GTSE1 and FGL1 was in the early time points of fibrosis. Immunofluorescence data also indicated that surrounding tissues of fibrotic regions showed overexpression of GTSE1 and FGL1, as well as irradiated fibrotic regions in mouse lungs, suggesting that upregulation of GTSE1 and FGL1 may occur during the process of going to fibrosis, but it is not an outcome.

In conclusion, we established a mouse irradiation system and identified the molecular signatures for IR-induced lung fibrosis. Although further functional analyses are required to determine the roles of GTSE1 or FGL1 during development of lung fibrosis, our results might provide the information that is useful for understanding IR-induced lung fibrosis.

## Electronic supplementary material

ESM 1 (DOCX 26 kb)

ESM 2 (PPTX 3499 kb)
