# Supplementary material for: Identification of molecular signatures involved in radiation-induced lung fibrosis
Source: J Mol Med (Berl). 2018 Nov 7;97(1):37–47. doi: 10.1007/s00109-018-1715-9 (PMC6326977; doi:10.1007/s00109-018-1715-9)
Supplement: Supplementary file 1 — (DOCX 26 kb) [file 109_2018_1715_MOESM1_ESM.docx]

**Materials and methods**

**Hydroxypoline and Ki67 staining**

Lung tissues were formalin-fixed, paraffin-embedded, and sections were prepared. Immunohistochemistry was performed using a VECTASTAIN Elite ABC Kit (Vector Laboratories Inc., Burlingame, CA, USA) using anti-hydroxyproline (Bioss, Woburn, MA, USA). Immunofluorescence staining was also performed using anti-Ki-67 (Dako, Glostrup, Denmark) antibody.

**Microarray experiment, RNA isolation and RT-PCR**

According to previously described methods [7], total RNA from the mouse lung tissues was prepared using the Easy-SpinTM total RNA extraction kit according to the manufacturer’s instructions (iNtRON Biotechnology, Seoul, Republic of Korea). Before performing the microarray experiment, the quality of the purified RNA was measured using the Agilent 2100 Bioanalyzer (Agilent Technologies, Santa Clara, CA, USA); only samples with an RNA integrity number (RIN) greater than 7.0 were included in the microarray analysis. RNAs from 3 mice at each time point were pooled to exclude experimental bias. Isolated total RNA was amplified and labeled using the Low RNA Input Linear Amplification kit PLUS (Agilent Technologies) and then hybridized to a microarray containing approximately 44,000 probes (~ 21,600 unique genes), in accordance with the manufacturer's instructions (Agilent Mouse whole genome 44K, Agilent Technologies). The arrays were scanned using an Agilent DNA Microarray Scanner (Agilent Technologies). The dataset is available online at the Gene Expression Omnibus (http://www.ncbi.nlm.nih.gov/geo) under the ID number GSE60541.

Total RNA was isolated from L132 cell using TRIzol® reagent (Qiazen, Valencia, CA, USA). RNA purity and concentration were measured with a Nanodrop. RNA was reverse transcribed using a ReverTra Ace® qPCR RT Kit (TOYOBO, kita-ku, Osaka, Japan) following the manufacturer's protocol, and PCR was performed to assess expression of the candidate genes using primers designed.

**Flow cytometry for cell death**

For cell cycle analysis, the cells were fixed in 70% ethanol at −20°C for at least 18 h. The fixed cells were washed once with PBS-EDTA and resuspended in 1 ml of PBS. After the addition of 10 μl each of propidium iodide (5mg/ml) and RNase (10 mg/ml), the samples were incubated for 30 min at 37°C and analyzed using a FACScan flow cytometer (BD Biosciences, CA, USA).

**Supplementary Figure Legends**

**Supplementary Fig. S1:** Morphologic observation after focal high dose radiation (90 Gy). **(a)** Representative gross findings. Mice were sacrificed at the indicated time points after irradiation and the lungs were photographed after complete fixation. **(b)** Isolation of fibrotic and neighboring regions from lung tissues after irradiation.

**Supplementary Fig. S2:** Histological changes in irradiated lungs within each time-point following irradiation. Sections were stained with hematoxylin and eosin after **(a)** focal high dose radiation (90 Gy) and **(b)** diffused radiation (20 Gy). Immunohistochemistry analysis for hydroxyproline in lung tissues at each time point after **(c)** 90 Gy and **(d)** 20 Gy. The arrows indicate the focally irradiated area. Magnification, x1.25 and x100; scale bar, 50 μm. All experimental lung tissue sections used three individual mouse samples.

**Supplementary Fig. S3:** Fibrosis related genes expression from the microarray data were compared after focal (90 Gy) and diffused (20 Gy) irradiation.

**Supplementary Fig. S4**: **(a)** TGFβ family gene expression patterns from the microarray data were compared after focal (90 Gy) and diffused (20 Gy) irradiation. **(b)** Microarray data of *gtse1* and *fgl1* for focal high-dose radiation (90 Gy) were compared to those for diffused (20 Gy) irradiation.

**Supplementary Fig. S5:** **(a)** Expression of GTSE1 and FGL1 in L132 cells was examined by RT-PCR (left) and western blotting (right) analysis after treatment of TGFβ (5 ng/ml) at indicated time points. **(b)** Western blot analysis in L132 cells after transfection of siRNA *gtse1* or *fgl1* with or without TGFβ (5 ng/ml) treatment. mRNA expression levels and protein expression levels were quantified using Image J software, and data are expressed as the fold change relative to each time point control or negative control (left). **(c)** Cellular morphology and **(d)** immunofluorescence co-staining of Fibronectin (green) and GTSE1 (red) or FGL1 (red) after 24 h of treatment of TGFβ (5 ng/ml) in A549 cells. Nuclear were counterstained with DAPI (blue). **(e)** Wound healing assays in A549 cells after transfection of siRNA *gtse1* or *fgl1* with or without TGFβ (5 ng/ml) treatment. Cell movement into wound was shown at 48 h post-scratch. Graphs represent relative wound width (mean ± SD*, *p<0.05* vs. corresponding control). All representative photomicrographs magnification, x100; scale bar, 50 μm.

**Supplementary Fig. S6:** Cell death using flow cytometric analysis after PI staining after siRNA *gtse1* or *fgl1* with or without radiation (8 Gy) or TGFβ (5 ng/ml) treatment in L132 cells.

**Supplementary Fig. S7:** Immunofluorescence staining of proliferation marker Ki-67 (green) and GTSE1 (red) in lung tissues at each time point. Nuclear were counterstained with DAPI. The arrows indicate the fibrosis area. Magnification, x100 and x200; scale bar, 50 μm. Lung tissue sections used three individual mouse samples. (F: focal irradiated region, N: neighboring region)

**Table S1.** Gene List used in RT-PCR and qRT-PCR analysis.

|  |  |
| --- | --- |
| **Gene name** | **Sequence** |
| Growth differentiation factor 15 (*gdf15*) | Forward: ACACTCAGGACACAAGCGACAT |
|  | Reverse: GACCCCAATCTCACCTCTGG |
| Carboxylesterases 5 (*ces5*) | Forward: GCACTACAAGTAGCACATT |
|  | Reverse: TGGTCAGCCTTCACATAG |
| G2 and S phase-expressed protein 1 (*gtse1*) | Forward: GCTTGCCTCTGACTTGCCTA |
|  | Reverse: CAAACGTTTCCTTGCCCTGG |
| Proline serine-rich coiled-coil protein 1 (*psrc1*) | Forward: TGAAGCGAGGTCTAAGCCGAC |
|  | Reverse: GTGCTCCAAGAACTCACGGT |
| Plasminogen activator, urokinase (*plau*) | Forward: CAGAACGGAGGTGTATGCGT |
|  | Reverse: TCTGGTAGATGGCTGCGAAC |
| Arylacetamide deacetylase (*aadac*) | Forward: TTACCTAAGACCTATATCATCACT |
|  | Reverse: AACTCCAACATTCTGAAGC |
| Cytoskeleton-associated protein 2 (*ckap2*) | Forward: CTTTGAGACCGACGTAGCGG |
|  | Reverse: GCACTCTTCTGGGGGCACAT |
| Centromere protein M (*cenpm*) | Forward: TGAACAGGGCTACCGTCTTG |
|  | Reverse: AAGCTTTCCACCCCCTGTGA |
| Spindle and kinetochore associated complex subunit 1 (*ska1*) | Forward: GTCTGGATTTGGCGTTTCGG |
|  | Reverse: AAGCGTGATTTTGCGCAGG |
| SHC SH2 domain-binding protein 1 (*shcbp1*) | Forward: GCAGGAGTTGTGGGTGGTAT |
|  | Reverse: GGTGGAGGAAACCACATGGA |
| Kinesin-like protein KIF2C (*kif2c*) | Forward: GCGCGCGGGATTTAAACTG |
|  | Reverse: CTGAGACAGTGGACATGCGA |
| PDZ binding kinase (*pbk*) | Forward: AGTTAGAACTCCAGTGCCCCT |
|  | Reverse: GCCATGTGCAAAGCAACTCT |
| Ubiquitin-conjugating enzyme E2 C (*ube2c*) | Forward: AAAGGAATCTCCGCCTTCCC |
|  | Reverse: TGCTGTGGGGTTTTTCCAGA |
| Fatty Acid-Binding Protein 1 (*fabp1*) | Forward: CGTGACTGAACTCAATGG |
|  | Reverse: TTCTCTTGCTGACTCTCTT |
| Repetin (*rptn*) | Forward: GGAACACTTGGACCGAGACA |
|  | Reverse: TCCGGCGTTCTCCTTCATAC |
| Keratin 13 (*krt13*) | Forward: CAGTCCCAGCTGAGCATGAA |
|  | Reverse: GGGACCCGTTGGAGGTAGTA |
| cystatin 9 (*cst9*) | Forward: TGGAGTTCTGGCTCCATGTC |
|  | Reverse: TACGTGACCAGTCCATGGGT |
| Asialoglycoprotein receptor 1 (*asgr1*) | Forward: ACATGGGCCCTGGATGGAC |
|  | Reverse: TCGCACGTCAGACACTAACT |
| Cell death-inducing DFFA-like effector c (*cidec*) | Forward: GTATTGCCAGGAGGCTGGG |
|  | Reverse: CTCCAAGCTGTGAGCCATGA |
| Protein phosphatase 1 regulatory inhibitor subunit 1B (*ppp1r1b*) | Forward: AGAAGGCAGAGTGGGGGATA |
|  | Reverse: GAAAAGCATGGCAGGGGTTG |
| Calpain 13 (*capn13*) | Forward: ACTACAGTTCCAGGTATTCAA |
|  | Reverse: GGGCAGCACTTCTAAATG |
| Paraoxonase 1 (*pon1*) | Forward: CCCATACTTACGGTCCTGGG |
|  | Reverse: GTGCCAATCAGCAGTTTCCC |
| Fibrinogen like 1 (*fgl1*) | Forward: GACCTTGGAGGCAAGAAGCA |
|  | Reverse: TGACTAGCCCACCACTGTACT |
| neutrophilic granule protein (*ngp*) | Forward: TGCAGTCGAGAGGATACCCA |
|  | Reverse: AGGAGGGGCAGATCCTGAAT |
| Fibronectin 1 (FN1) | Forward: CAGTGGGAGACCTCGAGAAG |
|  | Reverse: TCCCTCGGAACATCAGAAAC |
| Matrix metallopeptidase 2 (MMP2) | Forward: CCCCATGAAGCCTTGTTTACC |
|  | Reverse: GTGTAGATCGGGGCCATCAG |
| Matrix metallopeptidase 12 (MMP12) | Forward: TCATGATGATTGTGTTCTTACAGGT |
|  | Reverse: GTACATCGGGCACTCCACAT |
| Twist family bHLH transcription factor 1 (TWIST1) | Forward: TTCAGACCCTCAAACTGGCG |
|  | Reverse: CTGGGAATCTCTGTCCACGG |
| Interleukin 1 beta (IL1B) | Forward: TGCCACCTTTTGACAGTGATG |
|  | Reverse: GGAGCCTGTAGTGCAGTTGT |
| Interleukin 6 (IL6) | Forward: CCCCAATTTCCAATGCTCTCC |
|  | Reverse: CGCACTAGGTTTGCCGAGTA |
| Interleukin 13 (IL13) | Forward: TGCCATCTACAGGACCCAGA |
|  | Reverse: CTCATTAGAAGGGGCCGTGG |
| GAPDH | Forward: GTGAGCCCCAGCCTTCTCCAT |
|  | Reverse: GGTCGGTGTGAACGGATTTG |
| qGAPDH | Forward: ACTGTGGTCATGAGCCCTTC |
|  | Reverse: GGGTGTGAACCACGAGAAAT |
