# Supplementary material for: Identification of molecular signatures involved in radiation-induced lung fibrosis
Source: J Mol Med (Berl). 2018 Nov 7;97(1):37–47. doi: 10.1007/s00109-018-1715-9 (PMC6326977; doi:10.1007/s00109-018-1715-9)
Supplement: Supplementary file 2 — (PPTX 3499 kb) [file 109_2018_1715_MOESM2_ESM.pptx]

## Slide 1
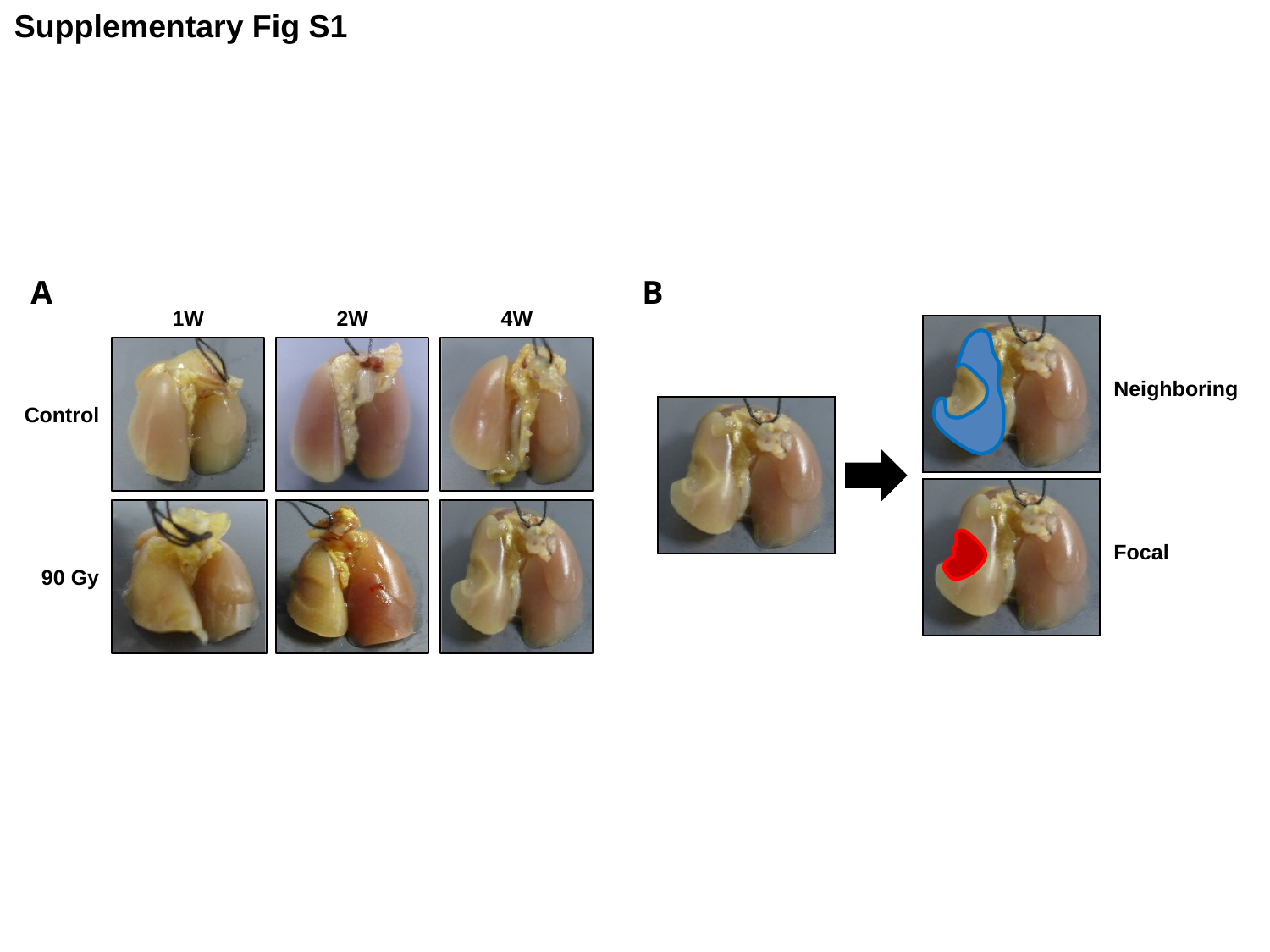

Supplementary Fig S1
A
B
1W
2W
4W
Control
90 Gy
Neighboring
Focal

## Slide 2
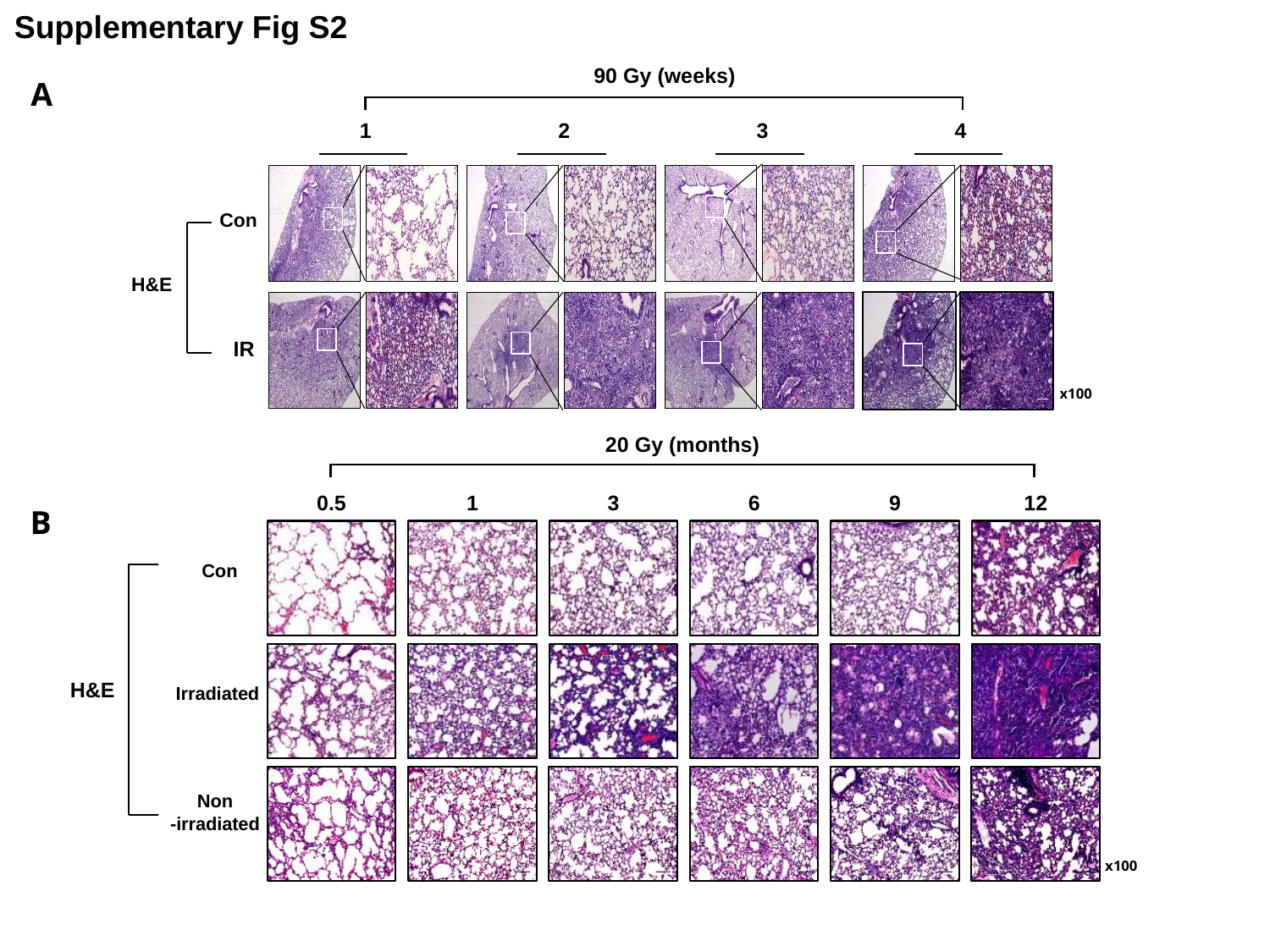

Supplementary Fig S2
90 Gy (weeks)
 1
 2
 3
 4
Con
H&E
IR
A
x100
20 Gy (months)
0.5
1
3
6
9
12
Con
H&E
Irradiated
Non
-irradiated
B
x100

## Slide 3
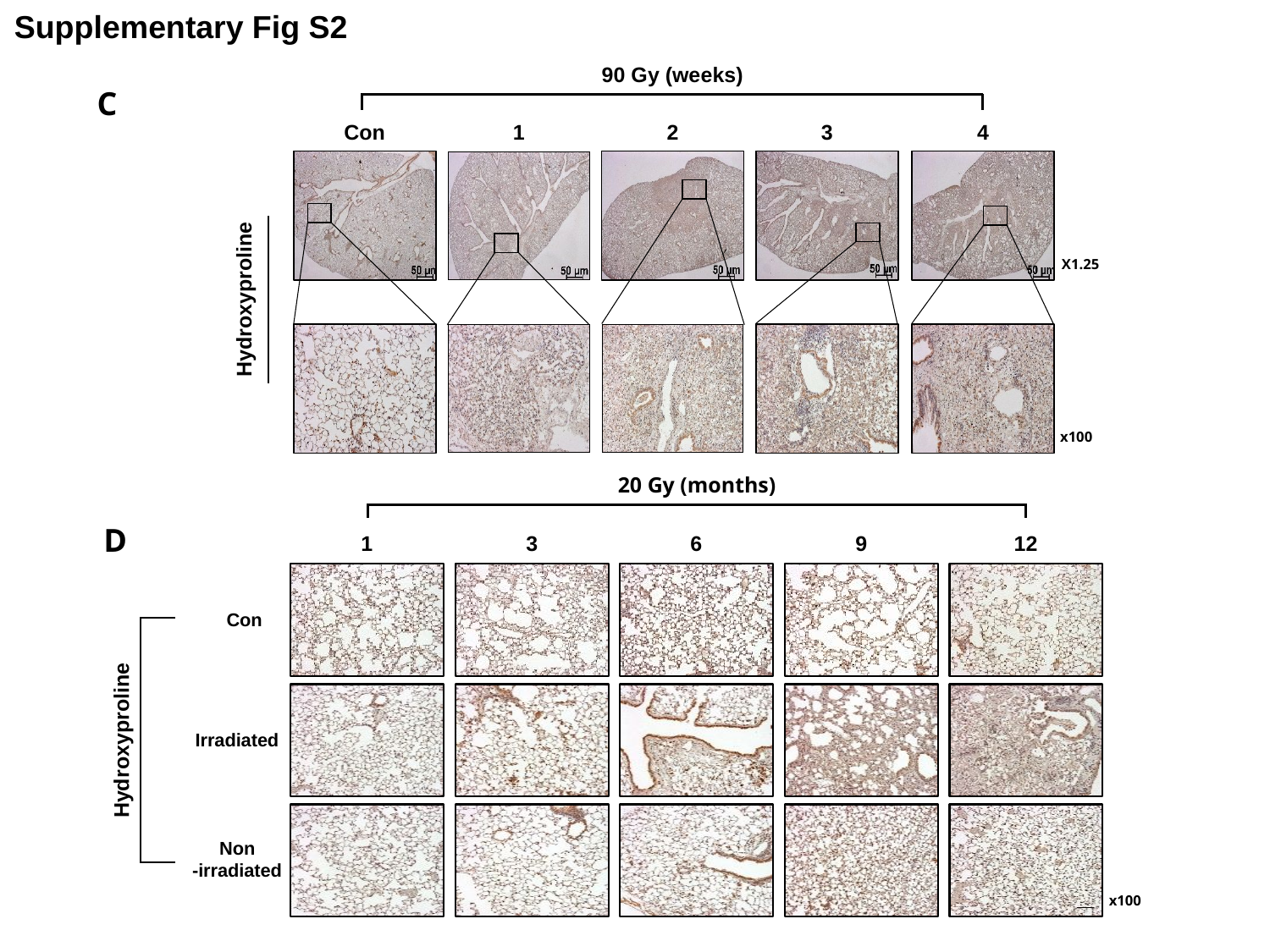

Supplementary Fig S2
90 Gy (weeks)
Con
1
2
3
4
X1.25
x100
Hydroxyproline
C
20 Gy (months)
D
1
3
6
9
12
Con
Hydroxyproline
Irradiated
Non
-irradiated
x100

## Slide 4
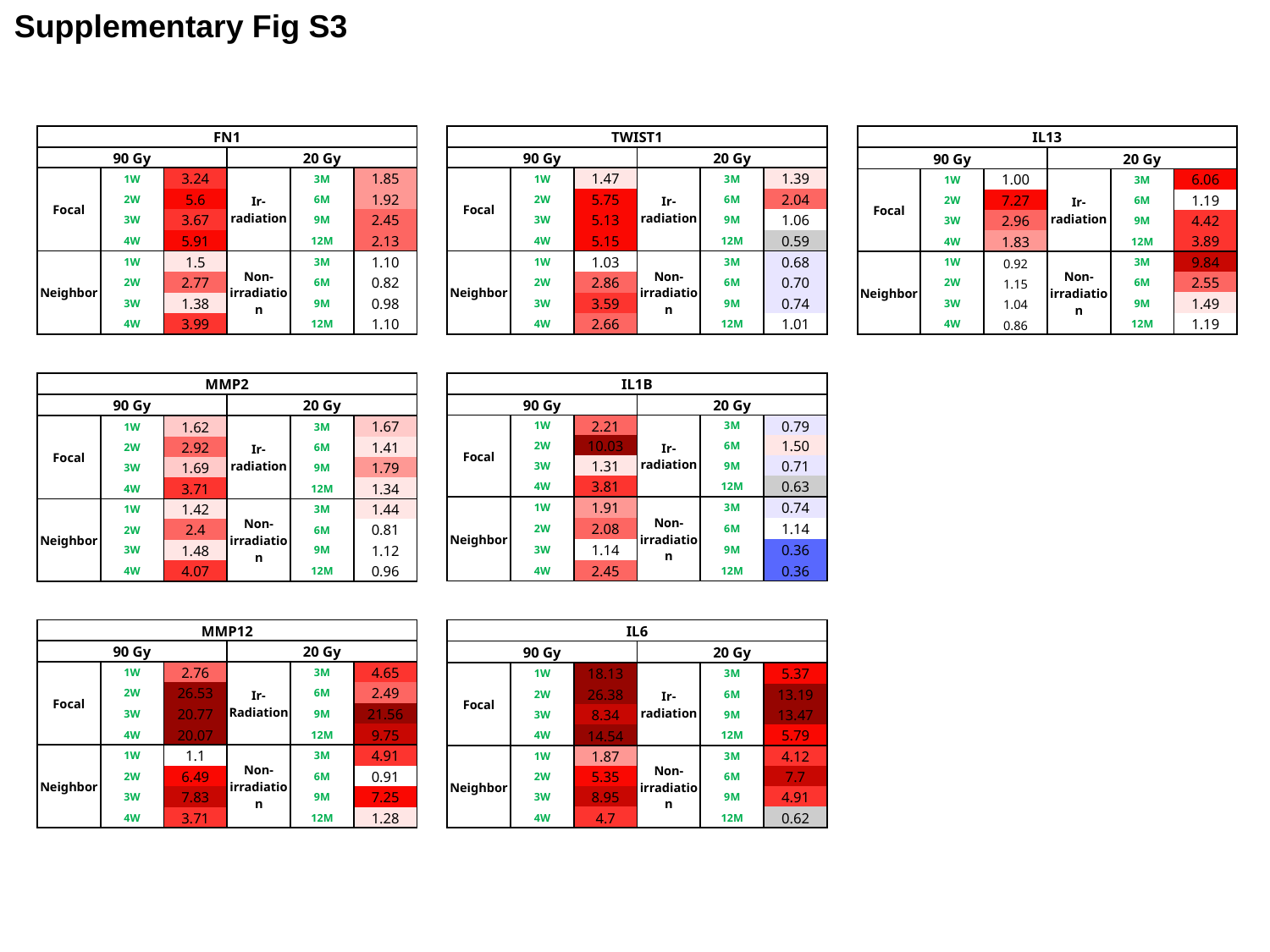

Supplementary Fig S3
| FN1 | | | | | |
| --- | --- | --- | --- | --- | --- |
| 90 Gy | | | 20 Gy | | |
| Focal | 1W | 3.24 | Ir- radiation | 3M | 1.85 |
| | 2W | 5.6 | | 6M | 1.92 |
| | 3W | 3.67 | | 9M | 2.45 |
| | 4W | 5.91 | | 12M | 2.13 |
| Neighbor | 1W | 1.5 | Non- irradiation | 3M | 1.10 |
| | 2W | 2.77 | | 6M | 0.82 |
| | 3W | 1.38 | | 9M | 0.98 |
| | 4W | 3.99 | | 12M | 1.10 |
| TWIST1 | | | | | |
| --- | --- | --- | --- | --- | --- |
| 90 Gy | | | 20 Gy | | |
| Focal | 1W | 1.47 | Ir- radiation | 3M | 1.39 |
| | 2W | 5.75 | | 6M | 2.04 |
| | 3W | 5.13 | | 9M | 1.06 |
| | 4W | 5.15 | | 12M | 0.59 |
| Neighbor | 1W | 1.03 | Non- irradiation | 3M | 0.68 |
| | 2W | 2.86 | | 6M | 0.70 |
| | 3W | 3.59 | | 9M | 0.74 |
| | 4W | 2.66 | | 12M | 1.01 |
| IL13 | | | | | |
| --- | --- | --- | --- | --- | --- |
| 90 Gy | | | 20 Gy | | |
| Focal | 1W | 1.00 | Ir- radiation | 3M | 6.06 |
| | 2W | 7.27 | | 6M | 1.19 |
| | 3W | 2.96 | | 9M | 4.42 |
| | 4W | 1.83 | | 12M | 3.89 |
| Neighbor | 1W | 0.92 | Non- irradiation | 3M | 9.84 |
| | 2W | 1.15 | | 6M | 2.55 |
| | 3W | 1.04 | | 9M | 1.49 |
| | 4W | 0.86 | | 12M | 1.19 |
| MMP2 | | | | | |
| --- | --- | --- | --- | --- | --- |
| 90 Gy | | | 20 Gy | | |
| Focal | 1W | 1.62 | Ir- radiation | 3M | 1.67 |
| | 2W | 2.92 | | 6M | 1.41 |
| | 3W | 1.69 | | 9M | 1.79 |
| | 4W | 3.71 | | 12M | 1.34 |
| Neighbor | 1W | 1.42 | Non-irradiation | 3M | 1.44 |
| | 2W | 2.4 | | 6M | 0.81 |
| | 3W | 1.48 | | 9M | 1.12 |
| | 4W | 4.07 | | 12M | 0.96 |
| IL1B | | | | | |
| --- | --- | --- | --- | --- | --- |
| 90 Gy | | | 20 Gy | | |
| Focal | 1W | 2.21 | Ir- radiation | 3M | 0.79 |
| | 2W | 10.03 | | 6M | 1.50 |
| | 3W | 1.31 | | 9M | 0.71 |
| | 4W | 3.81 | | 12M | 0.63 |
| Neighbor | 1W | 1.91 | Non- irradiation | 3M | 0.74 |
| | 2W | 2.08 | | 6M | 1.14 |
| | 3W | 1.14 | | 9M | 0.36 |
| | 4W | 2.45 | | 12M | 0.36 |
| MMP12 | | | | | |
| --- | --- | --- | --- | --- | --- |
| 90 Gy | | | 20 Gy | | |
| Focal | 1W | 2.76 | Ir- Radiation | 3M | 4.65 |
| | 2W | 26.53 | | 6M | 2.49 |
| | 3W | 20.77 | | 9M | 21.56 |
| | 4W | 20.07 | | 12M | 9.75 |
| Neighbor | 1W | 1.1 | Non- irradiation | 3M | 4.91 |
| | 2W | 6.49 | | 6M | 0.91 |
| | 3W | 7.83 | | 9M | 7.25 |
| | 4W | 3.71 | | 12M | 1.28 |
| IL6 | | | | | |
| --- | --- | --- | --- | --- | --- |
| 90 Gy | | | 20 Gy | | |
| Focal | 1W | 18.13 | Ir- radiation | 3M | 5.37 |
| | 2W | 26.38 | | 6M | 13.19 |
| | 3W | 8.34 | | 9M | 13.47 |
| | 4W | 14.54 | | 12M | 5.79 |
| Neighbor | 1W | 1.87 | Non- irradiation | 3M | 4.12 |
| | 2W | 5.35 | | 6M | 7.7 |
| | 3W | 8.95 | | 9M | 4.91 |
| | 4W | 4.7 | | 12M | 0.62 |

## Slide 5
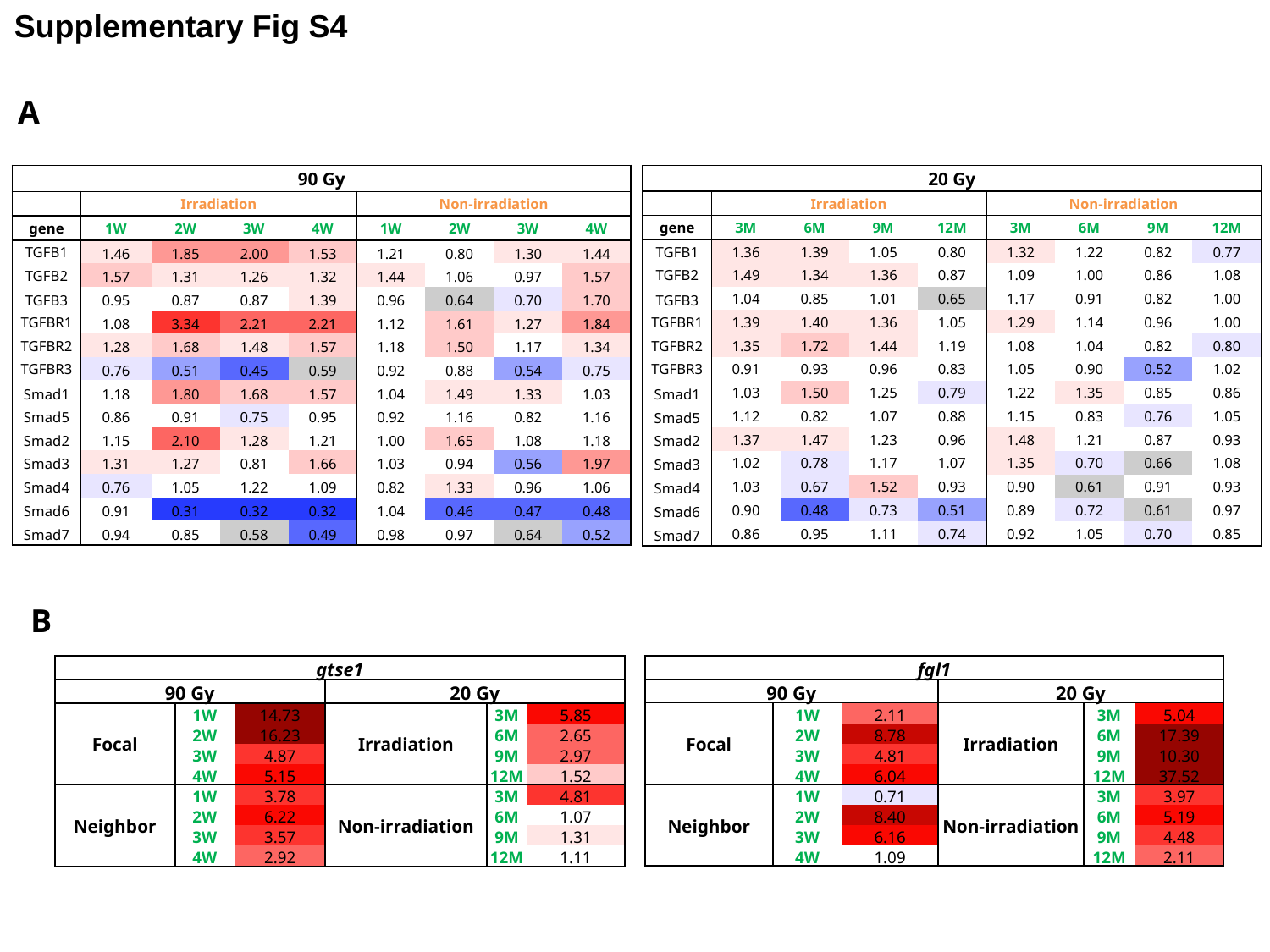

Supplementary Fig S4
A
| 90 Gy | | | | | | | | |
| --- | --- | --- | --- | --- | --- | --- | --- | --- |
| | Irradiation | | | | Non-irradiation | | | |
| gene | 1W | 2W | 3W | 4W | 1W | 2W | 3W | 4W |
| TGFB1 | 1.46 | 1.85 | 2.00 | 1.53 | 1.21 | 0.80 | 1.30 | 1.44 |
| TGFB2 | 1.57 | 1.31 | 1.26 | 1.32 | 1.44 | 1.06 | 0.97 | 1.57 |
| TGFB3 | 0.95 | 0.87 | 0.87 | 1.39 | 0.96 | 0.64 | 0.70 | 1.70 |
| TGFBR1 | 1.08 | 3.34 | 2.21 | 2.21 | 1.12 | 1.61 | 1.27 | 1.84 |
| TGFBR2 | 1.28 | 1.68 | 1.48 | 1.57 | 1.18 | 1.50 | 1.17 | 1.34 |
| TGFBR3 | 0.76 | 0.51 | 0.45 | 0.59 | 0.92 | 0.88 | 0.54 | 0.75 |
| Smad1 | 1.18 | 1.80 | 1.68 | 1.57 | 1.04 | 1.49 | 1.33 | 1.03 |
| Smad5 | 0.86 | 0.91 | 0.75 | 0.95 | 0.92 | 1.16 | 0.82 | 1.16 |
| Smad2 | 1.15 | 2.10 | 1.28 | 1.21 | 1.00 | 1.65 | 1.08 | 1.18 |
| Smad3 | 1.31 | 1.27 | 0.81 | 1.66 | 1.03 | 0.94 | 0.56 | 1.97 |
| Smad4 | 0.76 | 1.05 | 1.22 | 1.09 | 0.82 | 1.33 | 0.96 | 1.06 |
| Smad6 | 0.91 | 0.31 | 0.32 | 0.32 | 1.04 | 0.46 | 0.47 | 0.48 |
| Smad7 | 0.94 | 0.85 | 0.58 | 0.49 | 0.98 | 0.97 | 0.64 | 0.52 |
| 20 Gy | | | | | | | | |
| --- | --- | --- | --- | --- | --- | --- | --- | --- |
| | Irradiation | | | | Non-irradiation | | | |
| gene | 3M | 6M | 9M | 12M | 3M | 6M | 9M | 12M |
| TGFB1 | 1.36 | 1.39 | 1.05 | 0.80 | 1.32 | 1.22 | 0.82 | 0.77 |
| TGFB2 | 1.49 | 1.34 | 1.36 | 0.87 | 1.09 | 1.00 | 0.86 | 1.08 |
| TGFB3 | 1.04 | 0.85 | 1.01 | 0.65 | 1.17 | 0.91 | 0.82 | 1.00 |
| TGFBR1 | 1.39 | 1.40 | 1.36 | 1.05 | 1.29 | 1.14 | 0.96 | 1.00 |
| TGFBR2 | 1.35 | 1.72 | 1.44 | 1.19 | 1.08 | 1.04 | 0.82 | 0.80 |
| TGFBR3 | 0.91 | 0.93 | 0.96 | 0.83 | 1.05 | 0.90 | 0.52 | 1.02 |
| Smad1 | 1.03 | 1.50 | 1.25 | 0.79 | 1.22 | 1.35 | 0.85 | 0.86 |
| Smad5 | 1.12 | 0.82 | 1.07 | 0.88 | 1.15 | 0.83 | 0.76 | 1.05 |
| Smad2 | 1.37 | 1.47 | 1.23 | 0.96 | 1.48 | 1.21 | 0.87 | 0.93 |
| Smad3 | 1.02 | 0.78 | 1.17 | 1.07 | 1.35 | 0.70 | 0.66 | 1.08 |
| Smad4 | 1.03 | 0.67 | 1.52 | 0.93 | 0.90 | 0.61 | 0.91 | 0.93 |
| Smad6 | 0.90 | 0.48 | 0.73 | 0.51 | 0.89 | 0.72 | 0.61 | 0.97 |
| Smad7 | 0.86 | 0.95 | 1.11 | 0.74 | 0.92 | 1.05 | 0.70 | 0.85 |
B
| fgl1 | | | | | |
| --- | --- | --- | --- | --- | --- |
| 90 Gy | | | 20 Gy | | |
| Focal | 1W | 2.11 | Irradiation | 3M | 5.04 |
| | 2W | 8.78 | | 6M | 17.39 |
| | 3W | 4.81 | | 9M | 10.30 |
| | 4W | 6.04 | | 12M | 37.52 |
| Neighbor | 1W | 0.71 | Non-irradiation | 3M | 3.97 |
| | 2W | 8.40 | | 6M | 5.19 |
| | 3W | 6.16 | | 9M | 4.48 |
| | 4W | 1.09 | | 12M | 2.11 |
| gtse1 | | | | | |
| --- | --- | --- | --- | --- | --- |
| 90 Gy | | | 20 Gy | | |
| Focal | 1W | 14.73 | Irradiation | 3M | 5.85 |
| | 2W | 16.23 | | 6M | 2.65 |
| | 3W | 4.87 | | 9M | 2.97 |
| | 4W | 5.15 | | 12M | 1.52 |
| Neighbor | 1W | 3.78 | Non-irradiation | 3M | 4.81 |
| | 2W | 6.22 | | 6M | 1.07 |
| | 3W | 3.57 | | 9M | 1.31 |
| | 4W | 2.92 | | 12M | 1.11 |

## Slide 6
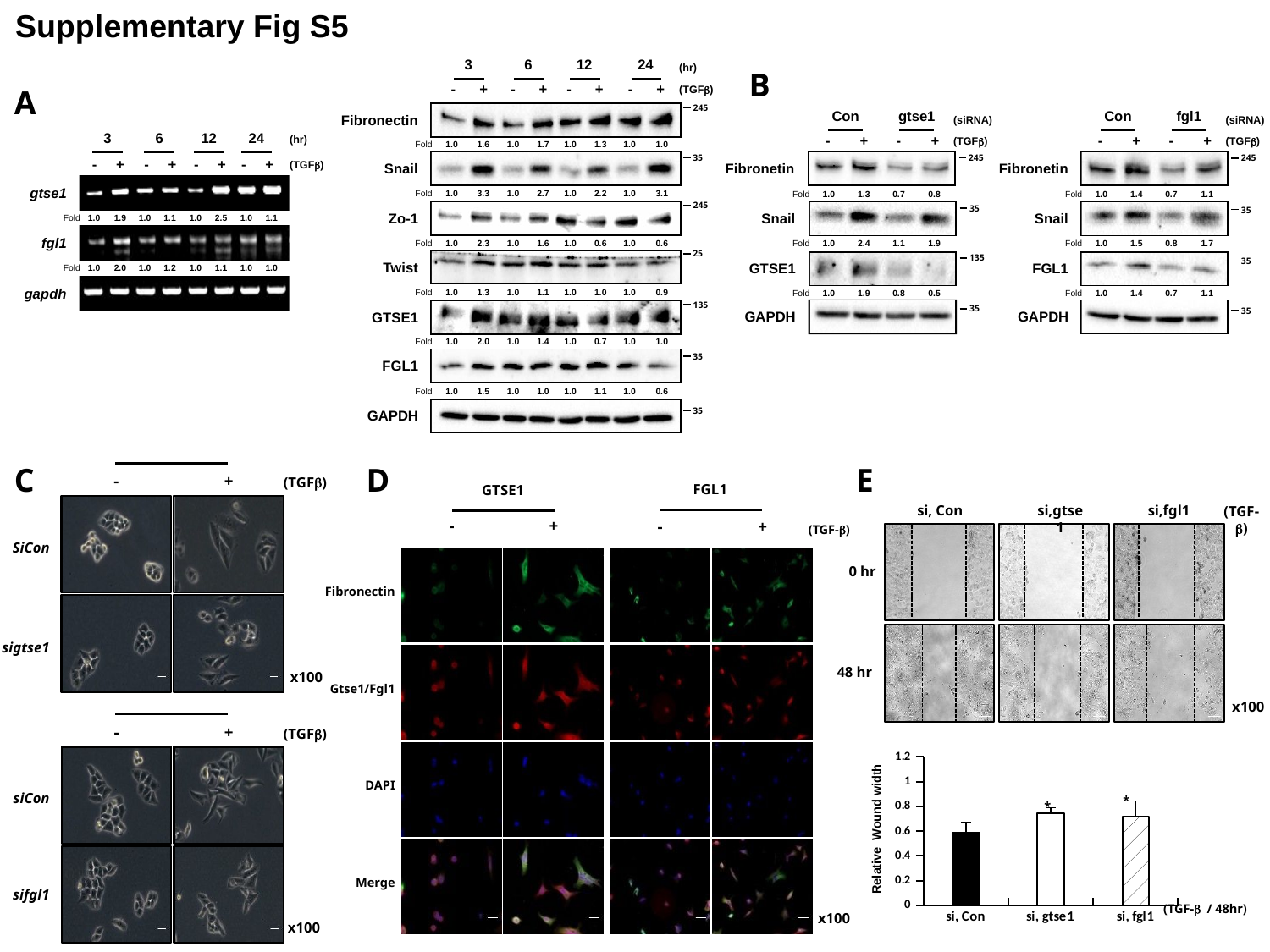

Supplementary Fig S5
6
-
+
12
-
+
24
-
+
3
-
+
(hr)
(TGFb)
245
Fibronectin
35
Snail
245
Zo-1
25
Twist
135
GTSE1
35
FGL1
35
GAPDH
Fold
1.0
1.6
1.0
1.7
1.0
1.3
1.0
1.0
Fold
1.0
3.3
1.0
2.7
1.0
2.2
1.0
3.1
Fold
1.0
2.3
1.0
1.6
1.0
0.6
1.0
0.6
Fold
1.0
1.3
1.0
1.1
1.0
1.0
1.0
0.9
Fold
1.0
2.0
1.0
1.4
1.0
0.7
1.0
1.0
Fold
1.0
1.5
1.0
1.0
1.0
1.1
1.0
0.6
B
A
Con
-
+
gtse1
-
+
(siRNA)
(TGFb)
245
Fibronetin
Fold
1.0
1.3
0.7
0.8
35
Snail
Fold
1.0
2.4
1.1
1.9
135
GTSE1
Fold
1.0
1.9
0.8
0.5
35
GAPDH
Con
-
+
fgl1
-
+
(siRNA)
(TGFb)
245
Fibronetin
Fold
1.0
1.4
0.7
1.1
35
Snail
Fold
1.0
1.5
0.8
1.7
35
FGL1
Fold
1.0
1.4
0.7
1.1
35
GAPDH
3
-
+
6
12
24
(hr)
-
+
-
+
-
+
(TGFb)
gtse1
Fold
1.0
1.9
1.0
1.1
1.0
2.5
1.0
1.1
fgl1
Fold
1.0
2.0
1.0
1.2
1.0
1.1
1.0
1.0
gapdh
C
D
E
-
+
(TGFb)
SiCon
sigtse1
-
+
(TGFb)
siCon
sifgl1
FGL1
-
+
(TGF-b)
GTSE1
-
+
Fibronectin
Gtse1/Fgl1
DAPI
Merge
si, Con
si,gtse1
si,fgl1
(TGF-b)
0 hr
48 hr
Relative Wound width
### Chart
| Category | |
|---|---|
| si, Con | 0.5896520861616861 |
| si, gtse1 | 0.7477789859472372 |
| si, fgl1 | 0.7180972875334238 |(TGF-b / 48hr)
*
*
x100
x100
x100
x100

## Slide 7
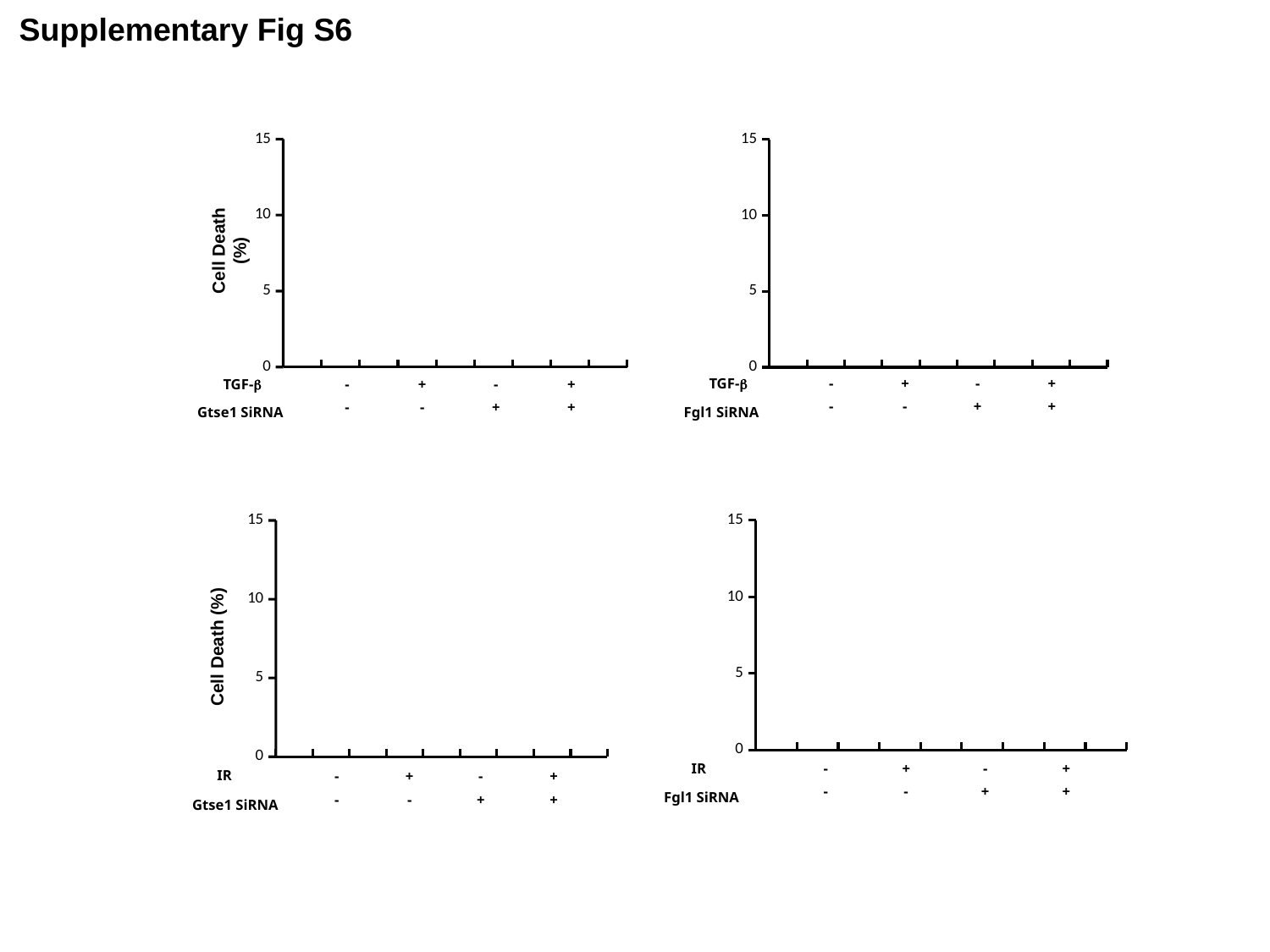

Supplementary Fig S6
### Chart
| Category | |
|---|---|
| Con si 24h | 7.77 |
| Con si 24h TGF-b | 6.37 |
| Gtse1 si 24h | 12.34 |
| Gtse1 si 24h TGF-b | 11.71 |Cell Death (%)
TGF-b
-
+
-
+
-
-
+
+
Gtse1 SiRNA
### Chart
| Category | |
|---|---|
| Con si 24h | 7.25 |
| Con si 24h TGF-b | 6.35 |
| Fgl si 24h | 8.91 |
| Fgl si 24h TGF-b | 8.969999999999999 |TGF-b
-
+
-
+
-
-
+
+
Fgl1 SiRNA
### Chart
| Category | |
|---|---|
| Con si 24h | 4.495 |
| Con si 24h TGF-b | 3.28 |
| Gtse1 si 24h | 4.4399999999999995 |
| Gtse1 si 24h TGF-b | 5.6850000000000005 |Cell Death (%)
IR
-
+
-
+
-
-
+
+
Gtse1 SiRNA
### Chart
| Category | |
|---|---|
| Con si 24h | 4.495 |
| Con si 24h TGF-b | 3.28 |
| Fgl si 24h | 5.535 |
| Fgl si 24h TGF-b | 7.875 |IR
-
+
-
+
-
-
+
+
Fgl1 SiRNA

## Slide 8
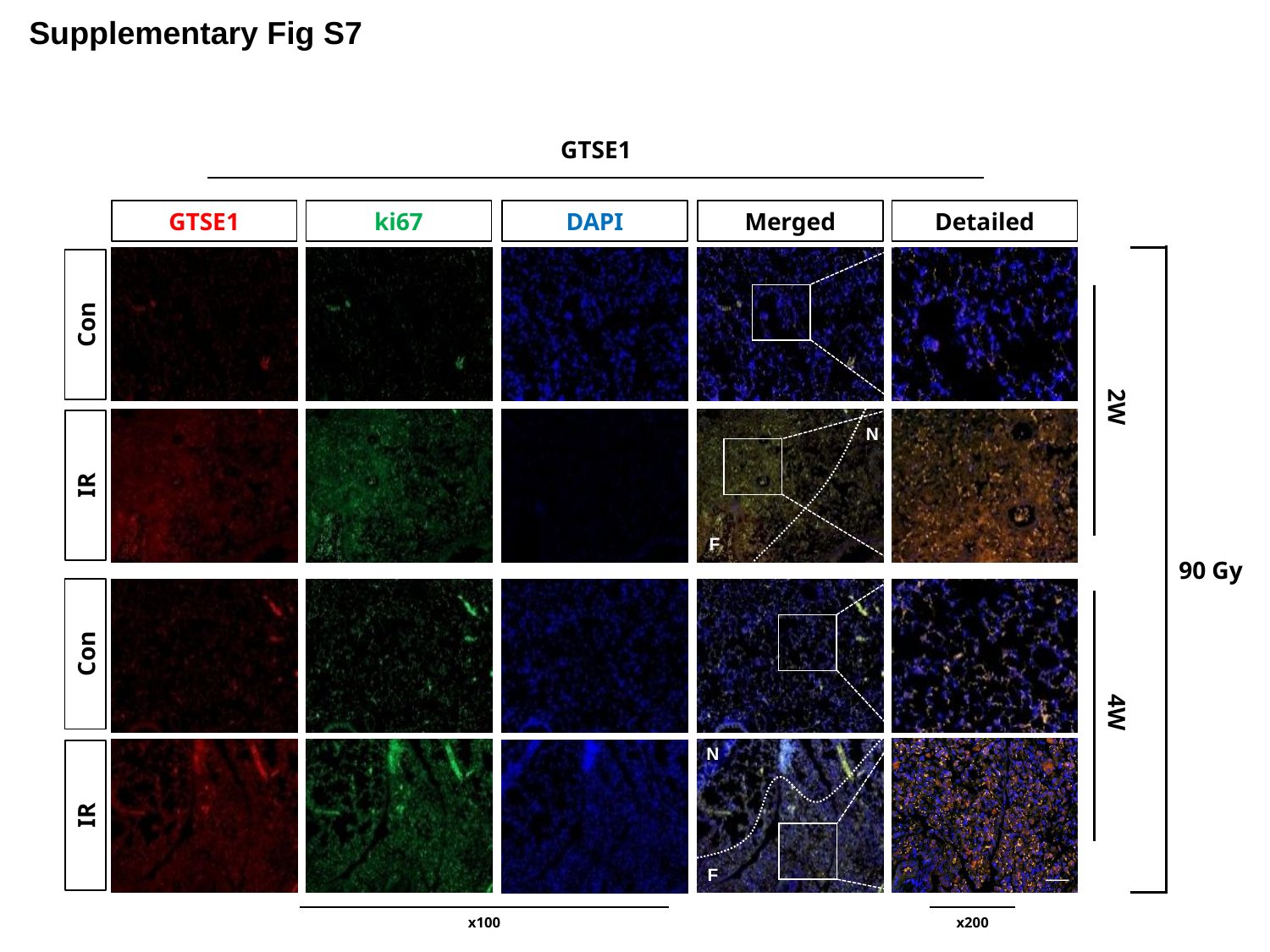

Supplementary Fig S7
GTSE1
GTSE1
ki67
DAPI
Merged
Detailed
90 Gy
2W
4W
Con
IR
Con
IR
x100
x200
N
F
N
F
